# Health-related quality of life and associated factors among primary caregivers of children with cerebral palsy, in Bahir Dar and Gondar cities, Ethiopia, 2022

**DOI:** 10.1371/journal.pone.0301050

**Published:** 2024-04-30

**Authors:** Tesfa Kassa, Hiruy Tadese, Getachew Azeze Eriku, Yohannes Abich, Molla Fentanew

**Affiliations:** Department of Physiotherapy, School of Medicine, College of Medicine and Health Science, University of Gondar Comprehensive Specialized Hospital, Gondar, Ethiopia; Wolkite University, ETHIOPIA

## Abstract

**Background:**

Caring for a child with cerebral palsy (CP) could negatively affect the Health-Related Quality of Life (HRQOL) of the mothers who are usually the primary caregivers. To the best of our ‘knowledge, there is a dearth of information on the HRQOL of primary caregivers of children with CP in Ethiopia. Therefore, this study aimed to investigate caregivers’ HRQOL and factors associated with it in Gondar and Bahir Dar Cities, Northwest Ethiopia, 2022.

**Methods:**

A community-based cross-sectional study was conducted among primary caregivers of Children with CP from April 20 to June 20, 2022, in Gondar and Bahir Dar cities. Convenience sampling was used to get study participants. Data were collected by trained health extension and community-based rehabilitation workers. The collected data were coded, cleaned, entered into EPI data, and exported to Stata-16 for analysis. A generalized linear model was employed to show the relationship between dependent and independent variables. A P-value≤0.05 was considered statistically significant at a 95% confidence interval.

**Result:**

In this study, HRQOL among primary caregivers of children with CP was 28.72(±13.38) and 23.26(±12.37) in the physical summary score (PSC) and mental summary score (MSC) respectively. Age 17-30yeas (p-value = 0.03), unable to read and write (p = 0.01), privately employed (p = 0.01) and government employed (p = 0.02), monthly income<1000 Ethiopian Birr (ETB) (p = 0.01), insufficient sleeping (p = 0.001), others relationship(p = 0.001), have three and above children (p = 0.001), others house composition (p = 0.003), have no helpers (p = 0.001) and third birth order of child (p = 0.03) were all factors associated with HRQOL in PSC. On the other hand, income<1000ETB (p = 0.05), insufficient sleeping (p = 0.001), others in relation to the child (p = 0.001), others in house composition (p = 0.03), dyskinetic CP (p = 0.01) and ataxic CP (p = 0.001) were all factors associated with HRQOL of caregivers in MSC components.

**Conclusion:**

The HRQOL among primary caregivers of children with CP in Bahir Dar and Gondar cities was low. Age, monthly income, educational status, sleeping status, relationship and house composition, number of living children, birth order of child, helpers, and type of CP were all significantly associated with HRQOL of primary caregivers of children with CP.

## Introduction

Constraints caused by Cerebral palsy (CP) includes motor dysfunction which is a hallmark of CP, speaking, intellectual and emotional problems [1–3]. It leads to dysfunction in self-care and make these children with CP dependent on others for their activities of daily living [4]. Those problems can result in demands and requirements for long-term care [5]. The role of the primary caregiver is essential in helping disabled individuals [6,7]. Because children with disabilities require more care, and their parents suffer more stress in taking care of them [8]. Taking care of these children, especially those who require special and long-term support, results in physical and mental stress for mothers [9,10].

The empirical evidence suggests that there is a wide variation in how caregivers adapt to the specific demands of care [11]. There are three aspects of circumstances that can cause stress and burden, which are characteristics of the affected person, the caregiver’s characteristics, and contextual factors. Those are important to understand the influence of these variables on the physical and mental health of the caregivers [9,12].

There is evidence to support the fact that the HRQOL of caregivers of children with CP influence the health outcome of the children with CP [13,14]. Caring for children with CP has a direct impact on caregivers’ lives and changes the caregiver’s normal routines [15].

As previously publish studies there are many factors associated with HRQOL of caregivers of children with CP. Among these age [16], educational status [16–18], occupation [19], monthly income [18,20,21], insufficient sleeping status [18], and relationship with child [21] were factors associated with HRQOL among primary caregivers.

Accordingly, caregivers HRQOL and the factors affecting it should be studied thoroughly to improve the care provided and the outcomes of children with CP and their families. Many factors interact together to shape the effect on the HRQOL of caregivers. To the best of our knowledge, there is a paucity of evidence on HRQOL among primary caregivers of children with CP in Ethiopia. Therefore, this study aimed to investigate the HRQOL and associated factors among primary caregivers of children with CP in Bahir Dar and Gondar cities, Northwest Ethiopia.

## Methods and materials

### Study design, period, and area

A community-based cross-sectional study was conducted to assess HRQOL and possible associated factors among primary caregivers of children with CP. The study studied from February to July 2022 and reported based on STROBE recommendations. The study was approved by ethical review committee of school of medicine and health science at University of Gondar, Ethiopia. Written informed consent were obtained from each participants and thumb impression were used participants who cannot read and write. This study was conducted in Bahir Dar and Gondar cities. Bahir Dar is the capital city of Amhara’s national regional state, located in the northwestern part of Ethiopia, at 565 km away from Addis Ababa. Gondar is located in the Amhara regional state, 738 km North West of Addis Ababa.

### Subjects and sampling technique

All primary caregivers of children with CP who lived in Bahir Dar and Gondar cities were the source population. Whereas, all primary caregivers of children with CP who lived in Bahir Dar and Gondar cities during the data collection period were the study population of the study. Participants aged 15 and above, both gender, and caregivers who care 6 month and above were included to the study. However, participants having a history of psychological disorders, antidepressant and anxiolytic drugs users, and caregivers with hearing and speech problems were excluded.

The study participants of this study were recruited by using a convenience sampling technique. Finally, with convenience sampling techniques 324 study participants were included in the study.

### Data collection tools and procedures

Primary Caregivers is the ones who spend most of the time with children with CP. The caregiver could be mothers, fathers, sisters, brothers, relatives, and employed workers. HRQOL of primary caregivers with CP was measured through **RAND** Short Form-36 (SF-36) health survey version 1.0 questionnaire. The scoring was demonstrated using norm-based scoring which is 50 as considered average; below 50 is considered below average and anything above 50 is considered above average. Therefore, lower scores in each dimension or summary score (physical component summary score and mental component summary score) are showing of poorer health-related quality of life in those domains and vice versa [22]. In addition to this, The Gross Motor Function Classification System (GMFCS), a 5-level classification system, were used to determine the severity levels of gross motor function of children and youth with CP.

The data were collected through face-to-face interviews by structured questionnaires, which has socio-demographic characteristics of caregivers (age, sex, marital status, level of education, occupation, monthly income, number of living children, relationship with the child, house composition, presence of an assistant, and sleeping status), socio-demographic and clinical characteristics of the child (sex, age, types of CP, type of CP based on involved body part, Type of disability, and severity level based on GMFCS), and RAND SF-36 health survey version 1.0 to determine health-related quality of life of primary caregivers. The questionnaire was first translated into Amharic language for data collection and translated back to English by a language expert. Health extension and community-based rehabilitation workers were the data collectors.

HRQOL of caregivers was assessed using the RAND SF-36 health survey version 1.0 questionnaire. The tool comprises 36 items classified with eight scales of domains. It includes Physical Functioning (PF), Role limitation due to Physical health problems (RP), Bodily Pain (BP), Role limitations due to Emotional problems (EP), General Health perception (GH), Social Functioning (SF), Vitality (VT) and Mental Health (MH). The scoring has two steps. Initially, pre-coded numeric values were recoded per the scoring manual. Second, items on the same scale were averaged together to make the scores. The higher the scores, the better the HRQOL. Finally, the tool sums up into two separate summary scores, which are physical summary score (PSC) and mental summary score (MSC) [23]. The validity and reliability of the tools have been demonstrated in Ethiopia [24].

### Data quality control and analysis

Prior to data collection, one-day training was provided for data collectors regarding the questionnaire of the study. The training was delivered by the principal investigators (HT and TK). Pre-tested were applied to around 5% of the total sample size of the study, which was performed at Debre Tabor referral hospital. Based on the finding of a pre-test the consistency and clarity of the questionnaire were checked. Furthermore, prior to data analysis, the principal investigator checked the completeness of the collected data.

The collected data were coded, entered into EPI data, and exported to STATA version 16 for analysis. Descriptive analysis such as frequency, percent, and mean (SD) of statistical findings was analyzed. A generalized linear model (GLM) with gamma family and link function was used to identify factors associated with HR-QOL. This model is able to handle large class errors of distributions. Variance inflation factor (VIF<10) was used to check multi-collinearity effect with independent factors. Independent factors with a 95% confidence level and P-value less than 0.05 in the final model were considered statistically significant and presented with an Adjusted Odds Ratio (AOR) with 95% CI.

### Ethical considerations

Ethical approval and clearance were obtained from the school of medicine ethical review committee (Ref.No: 470/04/2020) at University of Gondar College of medicine and health sciences.

Informed consent was obtained from each participant and the purposes and the importance of the study were explained to them. The Participant’s involvement in this study was voluntary and participants who were unwilling to participate in the study have the right withdrawal at any stage of the interview. Confidentiality was kept at all levels of the study. To ensure this, during data collection the study participants were identified using codes and unauthorized persons had no access to the collected data. The information that might expose the identity of the study participants was not collected. Computerized data were access only by principal investigator.

## Result

### Socio-demographic characteristics and other related factors of primary caregiver of children with cerebral palsy patients

A total of 324 primary caregivers of children with cerebral palsy in Gondar and Bahir Dar cities were participated in the study. Majority of primary caregivers were mothers 285(88%) and 121(62.7%) of caregivers do not have assistant or helper from family members to take care of their child with CP. The mean ages of caregivers were 35.4 years (±21.12) and only 6.8% of the participants have good sleeping status. Caregivers who have two children and did not perform regular exercise were 43.3% and 96.9% respectively (Table 1).

**Table 1:** Socio-demographic characteristics and other related factors of primary caregiver of children with cerebral palsy (n = 324) in 2022.

Variables		Frequency	Percentage (%)	Mean(SD)
Age	17–30 years	126	38.9	
	31–40 years	129	39.8	
	41–50 years	57	17.6	
	51 and above years	12	3.7	
Gender	Male	39	12	
	Female	285	88	
Marital status	Single	30	9.3	
	Married	91	28.1	
	Divorced	156	48.1	
	Widowed	47	14.5	
Educational level	Unable to read and write	150	46.3	
	Able to read and write	52	16	
	Primary school	24	7.4	
	Secondary school	45	13.9	
	Diploma/degree and above	53	16.4	
Occupation	Farmer	76	23.5	
	Merchant	19	5.9	
	Daily laborer	137	42.3	
	Private employed	44	13.6	
	Governmental employed	34	10.5	
	Other specific	14	4.3	
Monthly income	<1000 ETB	168	51.9	
	1000–4000 ETB	150	46.3	
	>4000 ETB	6	1.9	
Location of the participant	Gondar town	218	67.3	
	Bahir Dar town	106	32.7	
Sleeping status	Good	22	6.8	
	Bad	302	93.2	
What is your relationship with the child	Mother	285	88	
	Father	26	8	
	Other specific	13	4	
Number of living children	One	87	26.9	
	Two	140	43.2	
	Three and above	97	29.9	
What is your household composition?	Two parents together	90	27.8	
	Single mother	211	65.1	
	Single father	16	4.9	
	Others specific	7	2.2	
Do you have an assistant or helper from your family members for a child with CP?	Yes	121	37.3	
	No	203	62.7	
If yes, who mostly helps you?	Husband	42	32.3	
	Wife	14	10.8	
	Daughter	40	30.8	
	Son	6	4.6	
	Other specific	28	21.5	
Time devoted to diurnal childcare(Hours per day)				21.30±5.31
Regular exercise	Yes	10	3.1	
	No	314	96.9	

### Socio-demographic and clinical characteristics of children with cerebral palsy patients

Of the total, 66.7% of children with CP were male, among them 45.1% were the first child in the family with a mean age of 4.78 years (SD = 2.75). Regarding CP type, the majority of them were spastic (26.9%) followed by mixed type (25.0%). Multiple types of disability and physical disability in the type of disability were mostly experienced by participants 172(53.1%) and 110(34%) respectively (Table 2).

**Table 2:** Socio-demographic and clinical characteristics of children with cerebral palsy (n = 324) in 2022.

Variables		Frequency	Percentage (%)	Mean(SD)
Age (years)				1.33±0.47
Sex	Male	216	66.7	
	Female	108	33.3	
Birth order	First	146	45.1	
	Second	98	30.2	
	Third	58	17.9	
	Fourth	22	6.8	
Type of CP	Spastic	87	26.9	
	Athetoid	45	13.9	
	Ataxic	65	20.1	
	Hypotonic	46	14.2	
	Mixed	81	25.0	
Type of CP based on involved body part	Monoplegic	4	1.2	
	Hemiplegic	74	22.8	
	Diplegic	81	25.0	
	Tetraplegic	165	50.9	
Severity level (GMFCS)	Stage 1	30	9.3	
	Stage 2	80	24.7	
	Stage 3	153	47.2	
	Stage 4	59	18.2	
	Stage 5	2	6	
Type of disability	Physical	110	34.0	
	Intellectual/learning	34	10.5	
	Hearing	4	1.2	
	Vision	4	1.2	
	Multiple	172	53.1	

### Health-related quality of life among primary caregivers of children with CP

Out of the eight scales domains, physical functioning (50.09) and bodily pain (32.31) were the highest. Whereas, role limitation due to physical health (2.55) was the lowest. Moreover, PSC (28.72±13.38) score was higher than MSC (23.26±12.37).

The PSC and the MSC domains of HRQOL (skewness = 1.88 and kurtosis = 5.70) and (skewness = 1.51 and kurtosis = 4.93) had skewed distribution respectively.

### Factors associated with HRQOL among primary caregivers of children with CP patients

GLM was fitted to identify factors associated with HRQOL among primary caregivers of children with CP. Within this, we fitted two models comprising PSC (model 1) and MSC (model 2) as dependent variables.

#### Factors associated with Physical summary score

In the case of a model 1, around ten factors are associated with the Physical summary score. Seventeen to thirty years (exp(b):1.31, p-value = 0.028) and 31–40 years(exp(b): 1.28, p-value = 0.035) in the age of primary caregivers showed 31% and 28% times less HRQOL than 51 and above years old. Moreover, those unable to read and write (exp (b): 0.75, p-value = 0.001) and secondary school (exp(b): 0.80, p-value = 0.014) caregivers reported 25% and 20% poor HRQOL than those who had a diploma and above (Table 3).

**Table 3:** Factors associated with PSC in generalized linear model analysis (n = 324).

Variables		Exp(b)	95% CI	P-value
Age	17–30 years	1.31	1.03–1.66	**0.028**
	31–40 years	1.28	1.02–1.62	**0.035**
	41–50 years	1.19	.94–1.51	0.157
	51 and above	ref	ref	ref
Sex	Male	1.02	0.88–1.18	0.83
	Female	ref	ref	ref
Marital status	Single	0.96	.79–1.18	0.720
	Married	ref	ref	ref
	Divorced	0.99	.86–1.14	0.916
	Widowed	0.98	.83–1.16	0.821
Educational level	Unable to write and read	0.75	0.64–0.88	**0.001**
	Able to read and write	0.86	0.73–1.02	0.087
	Primary school	0.87	0.72–1.06	0.171
	Secondary school	0.80	0.68–0.96	**0.014**
	Diploma and above	ref	ref	ref
Occupation	Farmer	ref	ref	ref
	Merchant	1.01	0.84–1.21	0.08
	Daily laborer	0.89	0.78–1.01	0.082
	Private employed	0.79	0.67–0.94	**0.007**
	Governmental employed	0.78	0.63–0.96	**0.019**
	Others*	1.14	0.88–1.47	0.311
Monthly income	<1000 ETB	0.60	0.42–0.84	**0.003**
	1000–4000 ETB	0.68	0.49–0.95	**0.024**
	>4000 ETB	ref	ref	ref
Sleeping status	Good (sufficient)	ref	ref	ref
	Bad (insufficient)	0.72	0.61–0.86	**0.000**
Relationship with the child	Mother	ref	ref	ref
	Father	1.08	0.90–1.29	0.435
	Others**	0.51	0.37–0.71	**0.000**
Number of living children	One	ref	ref	ref
	Two	0.91	0.81–1.03	0.129
	Three and above	0.82	0.73–0.92	**0.001**
House composition	Two parents	ref	ref	ref
	Mother only	0.98	0.85–1.12	0.749
	Father only	0.89	0.70–1.14	0.366
	Others***	1.88	1.23–2.88	**0.003**
Do you have helpers from family members	Yes	ref	ref	ref
	No	0.82	0.75–0.91	**0.000**
Regular physical exercise	Yes	ref	ref	ref
	No	1.00	0.78–1.29	0.995
Birth order	One	ref	ref	ref
	Two	1.06	0.96–1.17	0.273
	Three	1.14	1.01–1.29	**0.032**
	Four	1.02	0.87–1.21	0.788
Type of CP	Spastic	ref	ref	ref
	Dyskinetic	1.09	0.95–1.24	0.206
	Ataxic	1.08	0.96–1.22	0.183
	Hypotonic	1.03	0.90–1.17	0.691
	Mixed	0.98	0.88–1.10	0.776
Severity in GMFCS	I	1.46	0.84–2.51	0.178
	II	1.47	0.85–2.53	0.164
	III	1.40	0.82–2.40	0.224
	IV	1.25	0.72–2.16	0.430
	V	ref	ref	ref
Type of disability	Physical	ref	ref	ref
	Intellectual	0.89	0.77–1.03	0.126
	Hearing	0.81	0.57–1.15	0.236
	Vision	1.26	0.86–1.84	0.237
	Mixed	0.96	0.88–1.05	0.337

* = (students and housemaid)

** = (sisters, brothers, employed workers), and

*** = (aunt, ankle, grandmother).

#### Factors associated with mental summary score

In the case of a model 2, around five variables are associated with mental summary scores these are low-paid monthly income, insufficient sleeping status, relationships with the child, house composition, and type of CP. Caregivers who paid <1000 (exp (b): 0.65; P-value = 0.046**)** had 35% less HRQOL than those who paid >4000 ETB. Likewise, caregivers who have paid 1000–4000 ETB (exp(b): 0.66; p-value = 0.045) had 34% less HRQOL than those who paid > 4000ETB.

Primary caregiver participants who had insufficient sleeping status (exp (b): 0.57; p-value = 0.000) had 43% less HRQOL than those who had sufficient sleep status. Moreover, others in caregivers relationship other than mothers and fathers (exp (b): 0.47; p-value = 0.000) were 53% less HRQOL than mothers (Table 4).

**Table 4:** Factors associated with MSC in generalized linear model analysis (n = 324).

Variables		Exp(b)	95% CI	P-value
Marital status	Single	0.86	0.67–1.09	0.220
	Married	ref	ref	ref
	Divorced	0.90	0.75–1.06	0.228
	Widowed	1.01	0.82–1.24	0.928
Educational level	Unable to write and read	0.92	0.75–1.11	0.352
	Able to read and write	1.06	0.86–1.29	0.623
	Primary school	0.83	0.65–1.04	0.097
	Secondary school	0.93	0.73–1.10	0.298
	Diploma and above	ref	ref	ref
Occupation	Farmer	ref	ref	ref
	Merchant	1.01	0.85–1.16	0.931
	Daily laborer	0.99	0.83–1.24	0.942
	Private employed	1.01	0.69–1.14	0.894
	Governmental employed	0.88	0.76–1.43	0.346
	Others*	1.04	0.79–1.50	0.789
Monthly income	<1000 ETB	0.65	0.43–0.99	**0.046**
	1000–4000 ETB	0.66	0.44–0.99	**0.045**
	>4000 ETB	ref	ref	ref
Sleeping status	Good (sufficient)	ref	ref	ref
	Bad (insufficient)	0.57	0.46–0.71	**0.000**
Relationship with the child	Mother	ref	ref	ref
	Father	0.87	0.71–1.05	0.152
	Others	0.47	0.32–0.70	**0.000**
House composition	Two parents	ref	ref	ref
	Mother only	0.94	0.78–1.11	0.451
	Father only	0.91	0.67–1.23	0.543
	Others**	1.76	1.05–2.94	**0.032**
Time devoted(hours per day)		0.99	0.98–1.01	0.569
Do you have helpers from family members	Yes	ref	ref	ref
	No	0.89	0.79–1.00	0.060
Physical exercise	Yes	ref	ref	ref
	No	1.03	0.76–1.41	0.791
Birth order	One	ref	ref	ref
	Two	0.97	0.86–1.08	0.526
	Three	1.06	0.90–1.18	0.640
	Four	0.97	0.80–1.17	0.731
Type of CP	Spastic	ref	ref	ref
	Dyskinetic	1.25	1.06–1.48	**0.005**
	Ataxic	1.31	1.13–1.51	**0.000**
	Hypotonic	1.17	0.99–1.35	0.070
	Mixed	1.06	0.92–1.20	0.444
Severity in GMFCS	I	1.77	0.96–3.29	0.069
	II	1.82	0.98–3.36	0.056
	III	1.75	0.95–3.21	0.072
	IV	1.35	0.73–2.53	0.337
	V	ref	ref	ref

* = (students and housemaid) and

** = (sisters, brothers, employed workers).

## Discussion

The main findings of this study were investigated through RAND SF-36 health survey version 1.0, which has eight domains. The scores range from the lowest physical health (2.55) to the highest score of physical functioning (50.09). PSC (28.72) was higher than MSC (23.26). Moreover, monthly income, sleeping status, house composition, and relationship with the child were associated factors with both PSC and MSC.

This study tried to investigate HRQOL among primary caregivers of children with cerebral palsy that was found that all of the eight scales were low. Particularly, role limitation due to physical health problems and role limitation due to emotional problems had the lowest scores. Our study, was higher than studies conducted in a rehabilitation center Khartoum-Sudan (overall 8.8) [19] and Ghana (median summary total score was 12.5) [25]. The plausible explanation could be that studies studied in those areas were used with small sample sizes. For example, a study in a rehabilitation center Khartoum-Sudan was conducted on 65 caregivers and in Ghana on 76 caregivers of participants. Beyond sample size differences, a study conducted in Ghana used a Pediatric Quality of Life Inventory Family Impact Module to determine caregivers’ quality of life.

In contrast, this study is lower than studies conducted at Selcuk University Medical Faculty(PSC; 55.8 and MSC: 52.31 scores) [26] and Edirne [27] in Turkey, Shanghai (PSC; 52.57 and MSC; 31.58) [21] and Anhui province of (PSC; 49.39 and MSC; 41.65) [16] in China, South Indian(mean mental HRQOL = 40.73 and mean score for physical HRQOL = 44.52) [20], Malaysia with a total impact score of 81.9 [18], and Brazil [28]. The possible explanation could be that those studies have different in characteristics of the participants, living standards of caregivers, and the quality of the service for CWD. For instance, in Shanghai China participants were recruited from the study who had well-organized rehabilitation services and governmental supported area for children with disabilities (CWD) and have higher standard living style.

Regarding factors associated with HRQOL on both PSC and MSC models were identified in this study. Caregivers who had less monthly income (<1000 and 1000–4000 ETB) had less HRQOL than caregivers who have paid >4000 ETB. Increased income could enhance the physical environment for parents who are caring for children and boost access to health services. As a result, this might give stable psychological and emotional feelings for caregivers. This study is supported by studies conducted in the Sarlahi and Rautahat Districts of Nepal [20,21] and Shanghai, China [21]. However, a study conducted in Malaysia reported that primary caregivers who have earned high monthly income had lower HRQOL than those who earned a low family monthly income.

Moreover, caregivers who had an insufficient sleeping status showed a significantly reduced HRQOL than caregivers who had a sufficient sleeping status. According to Elsayed and colleagues, children with CP experience sleeping issues more frequently than children with typical development [29]. Similarly, as reported by Wayte and colleagues, there had a strong connection between maternal depression and the child’s sleep problems [30]. This study also agreed with a study studied in Malaysia showed that insufficient or a problem in sleeping status had a less HRQOL of primary caregivers than those who had sufficient sleeping status [18].

Likewise, caregivers’ relationships with children of others (sister, brother, and employed care workers) had less HRQOL than mothers of the child on both physical and mental HRQOL. Furthermore, caregivers’ house composition of others (sister, brothers, aunts, ankles, grandmother) found lower HRQOL than those who had parents (both mothers and fathers) on both summary scores. On the other hand, 17–30 years, and 31–40 years old primary caregivers had 31% and 28% higher lower HRQOL than those who are 50 and above years old primary caregivers in physical summary scores respectively. The plausible explanation might be that being young age had highly vulnerable to fast fatigable tendency than the older age group to care children’s with cerebral palsy. On contrary, a study conducted in Tehran, Iran found that older age had poor HRQOL than the younger age group.

Another predictor associated with HRQOL were the educational status of caregivers. Being unable to read and write and secondary school of primary caregivers were 25% and 20% less HRQOL than those diploma and above caregivers in physical HRQOL respectively. The possible explanation might be that well-educated primary caregivers are believed to have a higher socioeconomic status. For this reason, caregivers who achieved up their educational status to diploma and above could have better HRQOL. The finding of this study is consistent with the studies conducted at Kelantan, Johor and Sarawak states in Malaysia [18], Anhui province of China [16], and Tehran, Iran [17]. Similarly, governmental and private employed primary caregivers had found poor HRQOL than farmers in physical HRQOL. This study agreed with a study done in Tehran, Iran [17]. They were reported that being unemployed had poor HRQOL than employed caregivers.

Being third birth order of a child had 14% reduced HRQOL of primary caregivers than those who are first birth order children in physical HRQOL. The result of this study is consistent with a study conducted in Shanghai, China [21]. On the other hand, primary caregivers who have three and above numbers of children had 18% less HRQOL than those who have a child in physical HRQOL. This might be due to the fact, caregivers need economical support and helpers to care for their children. So, as the number of children increases in a house, it may create an additional burden regarding caring them for primary caregivers.

Primary caregivers who had not an assistant or helper from the family members showed 18% less HRQOL than those who have an assistant or helpers in physical HRQOL. The plausible explanation is that when there are more family members, caregivers can receive physical and emotional support from other family members. Moreover, caregivers experience less psychological stress. This finding is agreed with a study studied in Shanghai, China [21].

According to Surender S and his colleagues, when compared to children with unilateral spastic CP, children with dyskinetic and bilateral spastic CP had lower HRQOL ratings, which is likely a result of the severity of the motor impairment in both conditions [31]. However, in our study, children with dyskinetic and ataxic types of CP had 25% and 31% less HRQOL of primary caregivers than those of children who had a spastic type of CP in mental HRQOL respectively.

The limitation of this study is lack of generalizability to all caregivers of children with CP because the study used a survey sampling method to recruit the study participants. In addition to this, the study focused only on two regional cities due to this caregivers who lived in rural areas were not addressed. Moreover, the cross-sectional nature of the study design does not show a causal-effect relationship of the possible associated factors with HRQOL.

## Conclusion

The HRQOL among primary caregivers of children with CP in the study area was low. Age, monthly income, educational status, sleeping status, relationship and house composition, number of living children, birth order of child, helpers, and type of CP were all significantly associated with HRQOL of primary caregivers of children with CP. Likewise, monthly income, sleeping status, relationship with child, and house composition were all significantly associated factors for both PSC and MSC domains of HRQOL of primary caregivers. Therefore, giving attentions to these factors affecting the HRQOL of primary caregivers of cerebral palsy will be necessary to improve the HRQOL of the caregivers.

## Supporting information

S1 Checklist *PLOS ONE* clinical studies checklist. (DOCX)

S1 Dataset (ZIP)
