# Supplementary material for: Health-related quality of life and associated factors among primary caregivers of children with cerebral palsy, in Bahir Dar and Gondar cities, Ethiopia, 2022
Source: PLoS One. 2024 Apr 30;19(4):e0301050. doi: 10.1371/journal.pone.0301050 (PMC11060524; doi:10.1371/journal.pone.0301050)
Supplement: Attachment — Submitted filename: Response to Reviewers.docx [file pone.0301050.s003.docx]

**Please see this comment before you will send to the reviewers.**

Comments suggested from reviewer #1 is not related to our documents as we observed from the comments. For example, Reviewer #1: Reviewers comment on mental health literacy among traditional healers toward mental illness in Northeast, Ethiopia: A mixed approach study; Which is not related to our topic since our topic is Health-related quality of life and associated factors among primary caregivers of children with cerebral palsy, in Bahir Dar and Gondar cities, Ethiopia

**Reviewers' comments:**

Reviewer's Responses to Questions

**Comments to the Author**

1. Is the manuscript technically sound, and do the data support the conclusions?

The manuscript must describe a technically sound piece of scientific research with data that supports the conclusions. Experiments must have been conducted rigorously, with appropriate controls, replication, and sample sizes. The conclusions must be drawn appropriately based on the data presented.

Reviewer #1: Yes

**Answer:** thank you so much

Reviewer #2: Yes

**Answer:** thank you so much

2. Has the statistical analysis been performed appropriately and rigorously?

Reviewer #1: Yes

**Answer:** thank you so much

Reviewer #2: Yes

**Answer:** thank you so much

3. Have the authors made all data underlying the findings in their manuscript fully available?

The [PLOS Data policy](http://www.plosone.org/static/policies.action#sharing) requires authors to make all data underlying the findings described in their manuscript fully available without restriction, with rare exception (please refer to the Data Availability Statement in the manuscript PDF file). The data should be provided as part of the manuscript or its supporting information, or deposited to a public repository. For example, in addition to summary statistics, the data points behind means, medians and variance measures should be available. If there are restrictions on publicly sharing data—e.g. participant privacy or use of data from a third party—those must be specified.

Reviewer #1: Yes

**Answer:** thank you so much

Reviewer #2: Yes

**Answer:** thank you so much

4. Is the manuscript presented in an intelligible fashion and written in standard English?

PLOS ONE does not copyedit accepted manuscripts, so the language in submitted articles must be clear, correct, and unambiguous. Any typographical or grammatical errors should be corrected at revision, so please note any specific errors here.

Reviewer #1: Yes

**Answer:** thank you so much

Reviewer #2: Yes

**Answer:** thank you so much

5. Review Comments to the Author

Please use the space provided to explain your answers to the questions above. You may also include additional comments for the author, including concerns about dual publication, research ethics, or publication ethics. (Please upload your review as an attachment if it exceeds 20,000 characters)

Reviewer #1: Reviewers comment on mental health literacy among traditional healers toward mental illness in Northeast, Ethiopia: A mixed approach study.
TITLE:-Satisfied and is area of researchable but I have a doubt with the study area since it only conducted in one city but generalized to northwest Ethiopia is not accepted.

**Answer:** thank you for your suggestion. I have incorporated in the documents. But our topic is ”Heath-related quality of life and associated factors among primary caregivers of children with cerebral palsy in Bahir Dar and Gondar cities, Ethiopia, 2022

ABSTRUCT
Background:-satisfied and well written but better to write about the global and national gabs about the mental literacy.

**Answer:** thank you for your suggestion. I have incorporated in the documents. But our topic is ”Heath-related quality of life and associated factors among primary caregivers of children with cerebral palsy in Bahir Dar and Gondar cities, Ethiopia, 2022 rather than mental literacy.
Method:-satisfied and well written but make bi variable instead of bivariate.

**Answer:** thank you for your comments. Still, the comments suggested for a different document.
Result:-Satisfied well written but there are findings that are not part of this study like **Traditional healers** provide a variety of explanations for the causes of mental illness, including biological, psychological, and supernatural ones.

**Answer:** thank you for your comments. Still, the comments suggested for a different document.
Conclusion: Satisfied and well written.

**Answer:** thank you so much
Keywords: Satisfied and well written

**Answer:** thank you so much
INTRODUCTION:-well written and satisfactory but some information needs updated information for instance in your background part Ethiopia, only has 60 psychiatrists and one mental health center, despite having approximately100 million people. In other words, for every psychiatrist, there are 1.67 million Ethiopians.

**Answer:** thank you for your comments. Still, the comments suggested for a different document.
METHOD AND MATERIALS
Study setting, design, and period:-clearly stated but please specify which type of study design you use.
Source of Population:-clearly stated

**Answer:** thank you so much
Study population:-clearly stated

**Answer:** thank you so much
Inclusion Criteria and Exclusion Criteria:-**why 6 month** is your exclusion criteria.

**Answer:** we have included study participants who are caring 6 month and above by referencing a similar study and caregivers who are caring 6 month and above might be affected their quality of life.
Sample size determination for quantitative:-clearly stated

**Answer:** thank you so much
Sample size determination for qualitative:-clearly stated

**Answer:** thank you so much
Sampling technique for quantitative:-clearly stated.

**Answer:** thank you so much
Sampling technique for qualitative: - not clearly stated. Make it brief and understandable.

**Answer:** thank you for your comments. Still, the comments suggested for a different document.
Data collection tools for quantitative and qualitative:-the data collection tool is standard tool but it needs clarification on the standard classification of the mental literacy based on the mean score.

**Answer:** thank you for your comments. Still, the comments suggested for a different document.
 What is the **average standard score**?

**Answer:** thank you for your comments. Still, the comments suggested for a different document.
Operational definitions:-clearly stated.

**Answer:** thank you so much
Data collection procedures for quantitative and qualitative:-satisfied and clearly stated but on qualitative data part what about the **recording materials**.

**Answer:** thank you for your comments. Still, the comments suggested for a different document.
Data processing and analysis:-The quantitative part was clearly stated but the **qualitative** part was not stated.

**Answer:** thank you for your comments. Still, the comments suggested for a different document.
Ethical Consideration:-clearly stated

**Answer:** thank you so much
RESULTS
Socio demographic Characteristics of Respondents:-clearly stated but the is **standard to describe variables as most, more and few.so consider that standard**

**Answer:** thank you for your comments. Still, the comments suggested for a different document.
Mental health experiences and social support of participants:-Clearly stated

**Answer:** thank you so much
Mental health literacy among **traditional healers:-**clearly stated but this is the qualitative part of analysis.

**Answer:** thank you for your comments. Still, the comments suggested for a different document.
Factors associated with mental health literacy among traditional healers:-

**Answer:** thank you for your comments. Still, the comments suggested for a different document.
Factors associated with mental health literacy among traditional healers:-clearly stated and well written.

**Answer:** thank you so much
DISCUSSION:-clearly stated and satisfactory.

**Answer:** thank you so much
LIMITATIONS OF THE STUDY:-clearly stated

**Answer:** thank you so much
CONCLUSION:-clearly stated

**Answer:** thank you so much

Reviewer #2: It is a technically correct paper and also the conclusion. What I miss and what should benefit is recommendations for improved quality of life for these mothers. Please provide some recommendations based on the results and discussion. **Please go through the format**. It is not correct. Good luck with your submission.

**Answer:** thank you so much for your comments. We have incorporated your comments to the document.

6. PLOS authors have the option to publish the peer review history of their article ([what does this mean?](https://journals.plos.org/plosone/s/editorial-and-peer-review-process#loc-peer-review-history)). If published, this will include your full peer review and any attached files.


If you choose “no”, your identity will remain anonymous but your review may still be made public.


**Do you want your identity to be public for this peer review?** For information about this choice, including consent withdrawal, please see our [Privacy Policy](https://www.plos.org/privacy-policy).

Reviewer #1: No

Reviewer #2: No

[NOTE: If reviewer comments were submitted as an attachment file, they will be attached to this email and accessible via the submission site. Please log into your account, locate the manuscript record, and check for the action link "View Attachments". If this link does not appear, there are no attachment files.]

While revising your submission, please upload your figure files to the Preflight Analysis and Conversion Engine (PACE) digital diagnostic tool, <https://pacev2.apexcovantage.com/>. PACE helps ensure that figures meet PLOS requirements. To use PACE, you must first register as a user. Registration is free. Then, login and navigate to the UPLOAD tab, where you will find detailed instructions on how to use the tool. If you encounter any issues or have any questions when using PACE, please email PLOS at [figures@plos.org](mailto:figures@plos.org). Please note that Supporting Information files do not need this step.
