# Supplementary material for: Health-related quality of life and associated factors among primary caregivers of children with cerebral palsy, in Bahir Dar and Gondar cities, Ethiopia, 2022
Source: PLoS One. 2024 Apr 30;19(4):e0301050. doi: 10.1371/journal.pone.0301050 (PMC11060524; doi:10.1371/journal.pone.0301050)
Supplement: Peer review history

# Peer review: Decision Letter 0

**DOI:** 10.1371/journal.pone.0301050.r001

**Authors:** Shegaw Tesfa Mengist

12 Oct 2023

PONE-D-23-18522Health-related quality of life and associated factors among primary caregivers of children with cerebral palsy, in Bahir Dar and Gondar cities, Ethiopia, 2022PLOS ONE

Dear Dr. Kassa,

Thank you for submitting your manuscript to PLOS ONE. After careful consideration, we feel that it has merit but does not fully meet PLOS ONE’s publication criteria as it currently stands. Therefore, we invite you to submit a revised version of the manuscript that addresses the points raised during the review process.

Please submit your revised manuscript by Nov 26 2023 11:59PM. If you will need more time than this to complete your revisions, please reply to this message or contact the journal office at plosone@plos.org. When you're ready to submit your revision, log on to https://www.editorialmanager.com/pone/ and select the 'Submissions Needing Revision' folder to locate your manuscript file.

Please include the following items when submitting your revised manuscript:

- A rebuttal letter that responds to each point raised by the academic editor and reviewer(s). You should upload this letter as a separate file labeled 'Response to Reviewers'.
- A marked-up copy of your manuscript that highlights changes made to the original version. You should upload this as a separate file labeled 'Revised Manuscript with Track Changes'.
- An unmarked version of your revised paper without tracked changes. You should upload this as a separate file labeled 'Manuscript'.

If you would like to make changes to your financial disclosure, please include your updated statement in your cover letter. Guidelines for resubmitting your figure files are available below the reviewer comments at the end of this letter.

If applicable, we recommend that you deposit your laboratory protocols in protocols.io to enhance the reproducibility of your results. Protocols.io assigns your protocol its own identifier (DOI) so that it can be cited independently in the future. For instructions see: https://journals.plos.org/plosone/s/submission-guidelines#loc-laboratory-protocols. Additionally, PLOS ONE offers an option for publishing peer-reviewed Lab Protocol articles, which describe protocols hosted on protocols.io. Read more information on sharing protocols at https://plos.org/protocols?utm_medium=editorial-email&utm_source=authorletters&utm_campaign=protocols.

We look forward to receiving your revised manuscript.

Kind regards,

Shegaw Tesfa Mengist

Academic Editor

PLOS ONE

Journal requirements:

When submitting your revision, we need you to address these additional requirements.

1. Please ensure that your manuscript meets PLOS ONE's style requirements, including those for file naming. The PLOS ONE style templates can be found at

https://journals.plos.org/plosone/s/file?id=wjVg/PLOSOne_formatting_sample_main_body.pdf and

https://journals.plos.org/plosone/s/file?id=ba62/PLOSOne_formatting_sample_title_authors_affiliations.pdf

2. PLOS requires an ORCID iD for the corresponding author in Editorial Manager on papers submitted after December 6th, 2016. Please ensure that you have an ORCID iD and that it is validated in Editorial Manager. To do this, go to ‘Update my Information’ (in the upper left-hand corner of the main menu), and click on the Fetch/Validate link next to the ORCID field. This will take you to the ORCID site and allow you to create a new iD or authenticate a pre-existing iD in Editorial Manager. Please see the following video for instructions on linking an ORCID iD to your Editorial Manager account: https://www.youtube.com/watch?v=_xcclfuvtxQ

3. Your ethics statement should only appear in the Methods section of your manuscript. If your ethics statement is written in any section besides the Methods, please move it to the Methods section and delete it from any other section. Please ensure that your ethics statement is included in your manuscript, as the ethics statement entered into the online submission form will not be published alongside your manuscript.

4. We are unable to open your Supporting Information file [Supporting information.rar]. Please kindly revise as necessary and re-upload.

5. Please review your reference list to ensure that it is complete and correct. If you have cited papers that have been retracted, please include the rationale for doing so in the manuscript text, or remove these references and replace them with relevant current references. Any changes to the reference list should be mentioned in the rebuttal letter that accompanies your revised manuscript. If you need to cite a retracted article, indicate the article’s retracted status in the References list and also include a citation and full reference for the retraction notice.

Reviewers' comments:

Reviewer's Responses to Questions

**Comments to the Author**

1. Is the manuscript technically sound, and do the data support the conclusions?

The manuscript must describe a technically sound piece of scientific research with data that supports the conclusions. Experiments must have been conducted rigorously, with appropriate controls, replication, and sample sizes. The conclusions must be drawn appropriately based on the data presented. 

Reviewer #1: Yes

Reviewer #2: Yes

**********

2. Has the statistical analysis been performed appropriately and rigorously? 

Reviewer #1: Yes

Reviewer #2: Yes

**********

3. Have the authors made all data underlying the findings in their manuscript fully available?

The PLOS Data policy requires authors to make all data underlying the findings described in their manuscript fully available without restriction, with rare exception (please refer to the Data Availability Statement in the manuscript PDF file). The data should be provided as part of the manuscript or its supporting information, or deposited to a public repository. For example, in addition to summary statistics, the data points behind means, medians and variance measures should be available. If there are restrictions on publicly sharing data—e.g. participant privacy or use of data from a third party—those must be specified.

Reviewer #1: Yes

Reviewer #2: Yes

**********

4. Is the manuscript presented in an intelligible fashion and written in standard English?

PLOS ONE does not copyedit accepted manuscripts, so the language in submitted articles must be clear, correct, and unambiguous. Any typographical or grammatical errors should be corrected at revision, so please note any specific errors here.

Reviewer #1: Yes

Reviewer #2: Yes

**********

5. Review Comments to the Author

Please use the space provided to explain your answers to the questions above. You may also include additional comments for the author, including concerns about dual publication, research ethics, or publication ethics. (Please upload your review as an attachment if it exceeds 20,000 characters)

Reviewer #1: Reviewers comment on mental health literacy among traditional healers toward mental illness in Northeast, Ethiopia: A mixed approach study.

TITLE:-Satisfied and is area of researchable but I have a doubt with the study area since it only conducted in one city but generalized to northwest Ethiopia is not accepted.

ABSTRUCT

Background:-satisfied and well written but better to write about the global and national gabs about the mental literacy.

Method:-satisfied and well written but make bi variable instead of bivariate.

Result:-Satisfied well written but there are findings that are not part of this study like Traditional healers provide a variety of explanations for the causes of mental illness, including biological, psychological, and supernatural ones.

Conclusion: Satisfied and well written.

Keywords: Satisfied and well written

INTRODUCTION:-well written and satisfactory but some information needs updated information for instance in your background part Ethiopia, only has 60 psychiatrists and one mental health center, despite having approximately100 million people. In other words, for every psychiatrist, there are 1.67 million Ethiopians.

METHOD AND MATERIALS

Study setting, design, and period:-clearly stated but please specify which type of study design you use.

Source of Population:-clearly stated

Study population:-clearly stated

Inclusion Criteria and Exclusion Criteria:-why 6 month is your exclusion criteria.

Sample size determination for quantitative:-clearly stated

Sample size determination for qualitative:-clearly stated

Sampling technique for quantitative:-clearly stated.

Sampling technique for qualitative: - not clearly stated. Make it brief and understandable.

Data collection tools for quantitative and qualitative:-the data collection tool is standard tool but it needs clarification on the standard classification of the mental literacy based on the mean score. What is the average standard score?

Operational definitions:-clearly stated.

Data collection procedures for quantitative and qualitative:-satisfied and clearly stated but on qualitative data part what about the recording materials.

Data processing and analysis:-The quantitative part was clearly stated but the qualitative part was not stated.

Ethical Consideration:-clearly stated

RESULTS

Socio demographic Characteristics of Respondents:-clearly stated but the is standard to describe variables as most, more and few.so consider that standard

Mental health experiences and social support of participants:-Clearly stated

Mental health literacy among traditional healers:-clearly stated but this is the qualitative part of analysis.

Factors associated with mental health literacy among traditional healers:-

Factors associated with mental health literacy among traditional healers:-clearly stated and well written.

DISCUSSION:-clearly stated and satisfactory.

LIMITATIONS OF THE STUDY:-clearly stated

CONCLUSION:-clearly stated

Reviewer #2: It is a technically correct paper and also the conclusion. What I miss and what should benefit is recommendations for improved quality of life for these mothers. Please provide som recommendations based on the results and discussion. Please go through the format. It is not correct. Good luck with your submission.

**********

6. PLOS authors have the option to publish the peer review history of their article (what does this mean?). If published, this will include your full peer review and any attached files.

If you choose “no”, your identity will remain anonymous but your review may still be made public.

**Do you want your identity to be public for this peer review?** For information about this choice, including consent withdrawal, please see our Privacy Policy.

Reviewer #1: No

Reviewer #2: No

**********

[NOTE: If reviewer comments were submitted as an attachment file, they will be attached to this email and accessible via the submission site. Please log into your account, locate the manuscript record, and check for the action link "View Attachments". If this link does not appear, there are no attachment files.]

While revising your submission, please upload your figure files to the Preflight Analysis and Conversion Engine (PACE) digital diagnostic tool, https://pacev2.apexcovantage.com/. PACE helps ensure that figures meet PLOS requirements. To use PACE, you must first register as a user. Registration is free. Then, login and navigate to the UPLOAD tab, where you will find detailed instructions on how to use the tool. If you encounter any issues or have any questions when using PACE, please email PLOS at figures@plos.org. Please note that Supporting Information files do not need this step.

# Author comment: Author response to Decision Letter 0

**DOI:** 10.1371/journal.pone.0301050.r002

26 Oct 2023

Please see this comment before you will send to the reviewers. 

Comments suggested from reviewer #1 is not related to our documents as we observed from the comments. For example, Reviewer #1: Reviewers comment on mental health literacy among traditional healers toward mental illness in Northeast, Ethiopia: A mixed approach study; Which is not related to our topic since our topic is Health-related quality of life and associated factors among primary caregivers of children with cerebral palsy, in Bahir Dar and Gondar cities, Ethiopia

Reviewers' comments:

Reviewer's Responses to Questions

Comments to the Author

1. Is the manuscript technically sound, and do the data support the conclusions?

The manuscript must describe a technically sound piece of scientific research with data that supports the conclusions. Experiments must have been conducted rigorously, with appropriate controls, replication, and sample sizes. The conclusions must be drawn appropriately based on the data presented.

Reviewer #1: Yes

Answer: thank you so much

Reviewer #2: Yes

Answer: thank you so much

________________________________________

2. Has the statistical analysis been performed appropriately and rigorously?

Reviewer #1: Yes

Answer: thank you so much

Reviewer #2: Yes

Answer: thank you so much

________________________________________

3. Have the authors made all data underlying the findings in their manuscript fully available?

The PLOS Data policy requires authors to make all data underlying the findings described in their manuscript fully available without restriction, with rare exception (please refer to the Data Availability Statement in the manuscript PDF file). The data should be provided as part of the manuscript or its supporting information, or deposited to a public repository. For example, in addition to summary statistics, the data points behind means, medians and variance measures should be available. If there are restrictions on publicly sharing data—e.g. participant privacy or use of data from a third party—those must be specified.

Reviewer #1: Yes

Answer: thank you so much

Reviewer #2: Yes

Answer: thank you so much

________________________________________

4. Is the manuscript presented in an intelligible fashion and written in standard English?

PLOS ONE does not copyedit accepted manuscripts, so the language in submitted articles must be clear, correct, and unambiguous. Any typographical or grammatical errors should be corrected at revision, so please note any specific errors here.

Reviewer #1: Yes

Answer: thank you so much

Reviewer #2: Yes

Answer: thank you so much

________________________________________

5. Review Comments to the Author

Please use the space provided to explain your answers to the questions above. You may also include additional comments for the author, including concerns about dual publication, research ethics, or publication ethics. (Please upload your review as an attachment if it exceeds 20,000 characters)

Reviewer #1: Reviewers comment on mental health literacy among traditional healers toward mental illness in Northeast, Ethiopia: A mixed approach study.

TITLE:-Satisfied and is area of researchable but I have a doubt with the study area since it only conducted in one city but generalized to northwest Ethiopia is not accepted.

Answer: thank you for your suggestion. I have incorporated in the documents. But our topic is ”Heath-related quality of life and associated factors among primary caregivers of children with cerebral palsy in Bahir Dar and Gondar cities, Ethiopia, 2022

________________________________________

ABSTRUCT

Background:-satisfied and well written but better to write about the global and national gabs about the mental literacy.

Answer: thank you for your suggestion. I have incorporated in the documents. But our topic is ”Heath-related quality of life and associated factors among primary caregivers of children with cerebral palsy in Bahir Dar and Gondar cities, Ethiopia, 2022 rather than mental literacy. 

Method:-satisfied and well written but make bi variable instead of bivariate.

Answer: thank you for your comments. Still, the comments suggested for a different document. 

Result:-Satisfied well written but there are findings that are not part of this study like Traditional healers provide a variety of explanations for the causes of mental illness, including biological, psychological, and supernatural ones.

Answer: thank you for your comments. Still, the comments suggested for a different document.

Conclusion: Satisfied and well written.

Answer: thank you so much

Keywords: Satisfied and well written

Answer: thank you so much 

INTRODUCTION:-well written and satisfactory but some information needs updated information for instance in your background part Ethiopia, only has 60 psychiatrists and one mental health center, despite having approximately100 million people. In other words, for every psychiatrist, there are 1.67 million Ethiopians.

Answer: thank you for your comments. Still, the comments suggested for a different document.

METHOD AND MATERIALS

Study setting, design, and period:-clearly stated but please specify which type of study design you use.

Source of Population:-clearly stated

Answer: thank you so much

Study population:-clearly stated

Answer: thank you so much

Inclusion Criteria and Exclusion Criteria:-why 6 month is your exclusion criteria.

Answer: we have included study participants who are caring 6 month and above by referencing a similar study and caregivers who are caring 6 month and above might be affected their quality of life. 

Sample size determination for quantitative:-clearly stated

Answer: thank you so much

Sample size determination for qualitative:-clearly stated

Answer: thank you so much

Sampling technique for quantitative:-clearly stated.

Answer: thank you so much

Sampling technique for qualitative: - not clearly stated. Make it brief and understandable.

Answer: thank you for your comments. Still, the comments suggested for a different document.

Data collection tools for quantitative and qualitative:-the data collection tool is standard tool but it needs clarification on the standard classification of the mental literacy based on the mean score.

Answer: thank you for your comments. Still, the comments suggested for a different document.

 What is the average standard score?

Answer: thank you for your comments. Still, the comments suggested for a different document.

Operational definitions:-clearly stated.

Answer: thank you so much 

Data collection procedures for quantitative and qualitative:-satisfied and clearly stated but on qualitative data part what about the recording materials.

Answer: thank you for your comments. Still, the comments suggested for a different document.

Data processing and analysis:-The quantitative part was clearly stated but the qualitative part was not stated.

Answer: thank you for your comments. Still, the comments suggested for a different document.

Ethical Consideration:-clearly stated

Answer: thank you so much

RESULTS

Socio demographic Characteristics of Respondents:-clearly stated but the is standard to describe variables as most, more and few.so consider that standard

Answer: thank you for your comments. Still, the comments suggested for a different document.

Mental health experiences and social support of participants:-Clearly stated

Answer: thank you so much 

Mental health literacy among traditional healers:-clearly stated but this is the qualitative part of analysis.

Answer: thank you for your comments. Still, the comments suggested for a different document.

Factors associated with mental health literacy among traditional healers:-

Answer: thank you for your comments. Still, the comments suggested for a different document.

Factors associated with mental health literacy among traditional healers:-clearly stated and well written.

Answer: thank you so much 

DISCUSSION:-clearly stated and satisfactory.

Answer: thank you so much 

LIMITATIONS OF THE STUDY:-clearly stated

Answer: thank you so much 

CONCLUSION:-clearly stated

Answer: thank you so much 

Reviewer #2: It is a technically correct paper and also the conclusion. What I miss and what should benefit is recommendations for improved quality of life for these mothers. Please provide some recommendations based on the results and discussion. Please go through the format. It is not correct. Good luck with your submission.

Answer: thank you so much for your comments. We have incorporated your comments to the document. 

________________________________________

6. PLOS authors have the option to publish the peer review history of their article (what does this mean?). If published, this will include your full peer review and any attached files.

If you choose “no”, your identity will remain anonymous but your review may still be made public.

Do you want your identity to be public for this peer review? For information about this choice, including consent withdrawal, please see our Privacy Policy.

Reviewer #1: No

Reviewer #2: No

________________________________________

[NOTE: If reviewer comments were submitted as an attachment file, they will be attached to this email and accessible via the submission site. Please log into your account, locate the manuscript record, and check for the action link "View Attachments". If this link does not appear, there are no attachment files.]

While revising your submission, please upload your figure files to the Preflight Analysis and Conversion Engine (PACE) digital diagnostic tool, https://pacev2.apexcovantage.com/. PACE helps ensure that figures meet PLOS requirements. To use PACE, you must first register as a user. Registration is free. Then, login and navigate to the UPLOAD tab, where you will find detailed instructions on how to use the tool. If you encounter any issues or have any questions when using PACE, please email PLOS at figures@plos.org. Please note that Supporting Information files do not need this step.

Attachment Submitted filename: Response to Reviewers.docx

# Peer review: Decision Letter 1

**DOI:** 10.1371/journal.pone.0301050.r003

**Authors:** Shegaw Tesfa Mengist

24 Nov 2023

PONE-D-23-18522R1Health-related quality of life and associated factors among primary caregivers of children with cerebral palsy, in Bahir Dar and Gondar cities, Ethiopia, 2022PLOS ONE

Dear Dr. Kassa,

Thank you for submitting your manuscript to PLOS ONE. After careful consideration, we feel that it has merit but does not fully meet PLOS ONE’s publication criteria as it currently stands. Therefore, we invite you to submit a revised version of the manuscript that addresses the points raised during the review process.

==============================

Please submit your revised manuscript by Jan 08 2024 11:59PM. If you will need more time than this to complete your revisions, please reply to this message or contact the journal office at plosone@plos.org. When you're ready to submit your revision, log on to https://www.editorialmanager.com/pone/ and select the 'Submissions Needing Revision' folder to locate your manuscript file.

Please include the following items when submitting your revised manuscript:

- A rebuttal letter that responds to each point raised by the academic editor and reviewer(s). You should upload this letter as a separate file labeled 'Response to Reviewers'.
- A marked-up copy of your manuscript that highlights changes made to the original version. You should upload this as a separate file labeled 'Revised Manuscript with Track Changes'.
- An unmarked version of your revised paper without tracked changes. You should upload this as a separate file labeled 'Manuscript'.

If you would like to make changes to your financial disclosure, please include your updated statement in your cover letter. Guidelines for resubmitting your figure files are available below the reviewer comments at the end of this letter.

If applicable, we recommend that you deposit your laboratory protocols in protocols.io to enhance the reproducibility of your results. Protocols.io assigns your protocol its own identifier (DOI) so that it can be cited independently in the future. For instructions see: https://journals.plos.org/plosone/s/submission-guidelines#loc-laboratory-protocols. Additionally, PLOS ONE offers an option for publishing peer-reviewed Lab Protocol articles, which describe protocols hosted on protocols.io. Read more information on sharing protocols at https://plos.org/protocols?utm_medium=editorial-email&utm_source=authorletters&utm_campaign=protocols.

We look forward to receiving your revised manuscript.

Kind regards,

Shegaw Tesfa Mengist

Academic Editor

PLOS ONE

Journal Requirements:

Please review your reference list to ensure that it is complete and correct. If you have cited papers that have been retracted, please include the rationale for doing so in the manuscript text, or remove these references and replace them with relevant current references. Any changes to the reference list should be mentioned in the rebuttal letter that accompanies your revised manuscript. If you need to cite a retracted article, indicate the article’s retracted status in the References list and also include a citation and full reference for the retraction notice.

[Note: HTML markup is below. Please do not edit.]

Reviewers' comments:

Reviewer's Responses to Questions

**Comments to the Author**

1. If the authors have adequately addressed your comments raised in a previous round of review and you feel that this manuscript is now acceptable for publication, you may indicate that here to bypass the “Comments to the Author” section, enter your conflict of interest statement in the “Confidential to Editor” section, and submit your "Accept" recommendation.

Reviewer #1: All comments have been addressed

**********

2. Is the manuscript technically sound, and do the data support the conclusions?

The manuscript must describe a technically sound piece of scientific research with data that supports the conclusions. Experiments must have been conducted rigorously, with appropriate controls, replication, and sample sizes. The conclusions must be drawn appropriately based on the data presented. 

Reviewer #1: Yes

**********

3. Has the statistical analysis been performed appropriately and rigorously? 

Reviewer #1: Yes

**********

4. Have the authors made all data underlying the findings in their manuscript fully available?

The PLOS Data policy requires authors to make all data underlying the findings described in their manuscript fully available without restriction, with rare exception (please refer to the Data Availability Statement in the manuscript PDF file). The data should be provided as part of the manuscript or its supporting information, or deposited to a public repository. For example, in addition to summary statistics, the data points behind means, medians and variance measures should be available. If there are restrictions on publicly sharing data—e.g. participant privacy or use of data from a third party—those must be specified.

Reviewer #1: Yes

**********

5. Is the manuscript presented in an intelligible fashion and written in standard English?

PLOS ONE does not copyedit accepted manuscripts, so the language in submitted articles must be clear, correct, and unambiguous. Any typographical or grammatical errors should be corrected at revision, so please note any specific errors here.

Reviewer #1: Yes

**********

6. Review Comments to the Author

Please use the space provided to explain your answers to the questions above. You may also include additional comments for the author, including concerns about dual publication, research ethics, or publication ethics. (Please upload your review as an attachment if it exceeds 20,000 characters)

Reviewer #1: Title –is very attractive

Background

Abstract-please show the gap of the problem on the context of Ethiopia briefly and do not use abbreviation in this part.

Methodology-it is better to use other probability sampling methods rather than survey.

Result –it is better to describe the association by using 95% CI rather than p-value.

In general it is very important topic for an input for further research but the topic should address other sites and cites.

So I recommend this topic for publication with minor revision

**********

7. PLOS authors have the option to publish the peer review history of their article (what does this mean?). If published, this will include your full peer review and any attached files.

If you choose “no”, your identity will remain anonymous but your review may still be made public.

**Do you want your identity to be public for this peer review?** For information about this choice, including consent withdrawal, please see our Privacy Policy.

Reviewer #1: No

**********

[NOTE: If reviewer comments were submitted as an attachment file, they will be attached to this email and accessible via the submission site. Please log into your account, locate the manuscript record, and check for the action link "View Attachments". If this link does not appear, there are no attachment files.]

While revising your submission, please upload your figure files to the Preflight Analysis and Conversion Engine (PACE) digital diagnostic tool, https://pacev2.apexcovantage.com/. PACE helps ensure that figures meet PLOS requirements. To use PACE, you must first register as a user. Registration is free. Then, login and navigate to the UPLOAD tab, where you will find detailed instructions on how to use the tool. If you encounter any issues or have any questions when using PACE, please email PLOS at figures@plos.org. Please note that Supporting Information files do not need this step.

# Author comment: Author response to Decision Letter 1

**DOI:** 10.1371/journal.pone.0301050.r004

29 Nov 2023

Answers for reviewer's comment

Please review your reference list to ensure that it is complete and correct. If you have cited papers that have been retracted, please include the rationale for doing so in the manuscript text, or remove these references and replace them with relevant current references. Any changes to the reference list should be mentioned in the rebuttal letter that accompanies your revised manuscript. If you need to cite a retracted article, indicate the article’s retracted status in the References list and also include a citation and full reference for the retraction notice.

Answer: we have not cited papers that have been retracted. 

[Note: HTML markup is below. Please do not edit.]

Reviewers' comments:

Reviewer's Responses to Questions

Comments to the Author

1. If the authors have adequately addressed your comments raised in a previous round of review and you feel that this manuscript is now acceptable for publication, you may indicate that here to bypass the “Comments to the Author” section, enter your conflict of interest statement in the “Confidential to Editor” section, and submit your "Accept" recommendation.

Reviewer #1: All comments have been addressed

Answer: thank you so much

________________________________________

2. Is the manuscript technically sound, and do the data support the conclusions?

The manuscript must describe a technically sound piece of scientific research with data that supports the conclusions. Experiments must have been conducted rigorously, with appropriate controls, replication, and sample sizes. The conclusions must be drawn appropriately based on the data presented.

Reviewer #1: Yes

Answer: thank you so much

________________________________________

3. Has the statistical analysis been performed appropriately and rigorously?

Reviewer #1: Yes

Answer: thank you so much

________________________________________

4. Have the authors made all data underlying the findings in their manuscript fully available?

The PLOS Data policy requires authors to make all data underlying the findings described in their manuscript fully available without restriction, with rare exception (please refer to the Data Availability Statement in the manuscript PDF file). The data should be provided as part of the manuscript or its supporting information, or deposited to a public repository. For example, in addition to summary statistics, the data points behind means, medians and variance measures should be available. If there are restrictions on publicly sharing data—e.g. participant privacy or use of data from a third party—those must be specified.

Reviewer #1: Yes

Answer: thank you so much

________________________________________

5. Is the manuscript presented in an intelligible fashion and written in Standard English?

PLOS ONE does not copyedit accepted manuscripts, so the language in submitted articles must be clear, correct, and unambiguous. Any typographical or grammatical errors should be corrected at revision, so please note any specific errors here.

Reviewer #1: Yes

Answer: thank you so much

________________________________________

6. Review Comments to the Author

Please use the space provided to explain your answers to the questions above. You may also include additional comments for the author, including concerns about dual publication, research ethics, or publication ethics. (Please upload your review as an attachment if it exceeds 20,000 characters)

Reviewer #1: Title –is very attractive

Background

Abstract-please show the gap of the problem on the context of Ethiopia briefly and do not use abbreviation in this part.

Answer: thank you for your question and suggestions. We have incorporated it into the document. 

Methodology-it is better to use other probability sampling methods rather than survey.

Answer: thank you for your question. We have corrected it and incorporated it into the main document. 

Result –it is better to describe the association by using 95% CI rather than p-value.

Answer: thank you for your questions and suggestions. We have described the association based on 95% CI and p-value. 

In general it is very important topic for an input for further research but the topic should address other sites and cites.

So I recommend this topic for publication with minor revision

________________________________________

7. PLOS authors have the option to publish the peer review history of their article (what does this mean?). If published, this will include your full peer review and any attached files.

If you choose “no”, your identity will remain anonymous but your review may still be made public.

Do you want your identity to be public for this peer review? For information about this choice, including consent withdrawal, please see our Privacy Policy.

Reviewer #1: No

________________________________________

[NOTE: If reviewer comments were submitted as an attachment file, they will be attached to this email and accessible via the submission site. Please log into your account, locate the manuscript record, and check for the action link "View Attachments". If this link does not appear, there are no attachment files.]

While revising your submission, please upload your figure files to the Preflight Analysis and Conversion Engine (PACE) digital diagnostic tool, https://pacev2.apexcovantage.com/. PACE helps ensure that figures meet PLOS requirements. To use PACE, you must first register as a user. Registration is free. Then, login and navigate to the UPLOAD tab, where you will find detailed instructions on how to use the tool. If you encounter any issues or have any questions when using PACE, please email PLOS at figures@plos.org. Please note that Supporting Information files do not need this step.

Attachment Submitted filename: Response to Reviewers.docx

# Editor report: Decision Letter 2

**DOI:** 10.1371/journal.pone.0301050.r005

**Authors:** Shegaw Tesfa Mengist

11 Mar 2024

Health-related quality of life and associated factors among primary caregivers of children with cerebral palsy, in Bahir Dar and Gondar cities, Ethiopia, 2022

PONE-D-23-18522R2

Dear Authors,

We’re pleased to inform you that your manuscript has been judged scientifically suitable for publication and will be formally accepted for publication once it meets all outstanding technical requirements.

Within one week, you’ll receive an e-mail detailing the required amendments. When these have been addressed, you’ll receive a formal acceptance letter and your manuscript will be scheduled for publication.

An invoice will be generated when your article is formally accepted. Please note, if your institution has a publishing partnership with PLOS and your article meets the relevant criteria, all or part of your publication costs will be covered. Please make sure your user information is up-to-date by logging into Editorial Manager at http://www.editorialmanager.com/pone/ and clicking the ‘Update My Information' link at the top of the page. If you have any questions relating to publication charges, please contact our Author Billing department directly at authorbilling@plos.org.

If your institution or institutions have a press office, please notify them about your upcoming paper to help maximize its impact. If they’ll be preparing press materials, please inform our press team as soon as possible -- no later than 48 hours after receiving the formal acceptance. Your manuscript will remain under strict press embargo until 2 pm Eastern Time on the date of publication. For more information, please contact onepress@plos.org.

Kind regards,

Shegaw Tesfa Mengist

Academic Editor

PLOS ONE

Additional Editor Comments (optional):

All comments addressed by the Authors

Reviewers' comments:
